# Gastrointestinal Interoception in Eating Disorders: Charting a New Path

**DOI:** 10.1007/s11920-022-01318-3

**Published:** 2022-01-21

**Authors:** Sahib S. Khalsa, Laura A. Berner, Lisa M. Anderson

**Affiliations:** 1 Laureate Institute for Brain Research, 6655 South Yale Ave, Tulsa, OK 74136 USA; 2 Oxley College of Health Sciences, University of Tulsa, Tulsa, OK USA; 3 Department of Psychiatry, Icahn School of Medicine at Mount Sinai, New York, NY USA; 4 Department of Psychiatry and Behavioral Sciences, University of Minnesota Medical School, Minneapolis, MN USA

**Keywords:** Anorexia nervosa, Bulimia nervosa, Binge-eating disorder, Avoidant/restrictive food intake disorder, Interoceptive awareness, Digestion

## Abstract

**Purpose of Review:**

Abnormal interoception has been consistently observed across eating disorders despite limited inclusion in diagnostic conceptualization. Using the alimentary tract as well as recent developments in interoceptive neuroscience and predictive processing as a guide, the current review summarizes evidence of gastrointestinal interoceptive dysfunction in eating disorders.

**Recent Findings:**

Eating is a complex process that begins well before and ends well after food consumption. Abnormal prediction and prediction-error signals may occur at any stage, resulting in aberrant gastrointestinal interoception and dysregulated gut sensations in eating disorders. Several interoceptive technologies have recently become available that can be paired with computational modeling and clinical interventions to yield new insights into eating disorder pathophysiology.

**Summary:**

Illuminating the neurobiology of gastrointestinal interoception in eating disorders requires a new generation of studies combining experimental probes of gut physiology with computational modeling. The application of such techniques within clinical trials frameworks may yield new tools and treatments with transdiagnostic relevance.

## Introduction

Eating disorders are psychiatric conditions characterized by aberrant eating and compensatory behavior patterns that are associated with severe medical complications, psychological comorbidities, and increased mortality [1]. Neurobiological models of eating disorders commonly emphasize the role of interactions among psychological traits and various cognitive functions (e.g., cognitive control, habit-learning), value processing (e.g., reward learning), and affective functioning (e.g., fear learning/generalization) [2–6]. Although interoceptive signaling is often linked with these processes, less attention is paid to the role of interoception.

The current review critically re-evaluates the role of interoception in eating disorders, with a focus on gastrointestinal interoception. It is organized around the potential points of altered interoception throughout the gastrointestinal tract and considers the associated implications for eating disorders.^1^ While prior reviews have touched on the role of interoception in eating disorders [7–9], gastrointestinal symptoms [10, 11], or related processes such as hunger/thirst [12], here we emphasize the importance of understanding gastrointestinal interoception through the lens of predictive processing, whereby the nervous system is engaged in predicting upcoming states in relation to current states, and refining these predictions via error signaling. Finally, we highlight several methodological developments relevant to the study of gastrointestinal interoception and discuss their implications for advancing the clinical understanding and treatment of eating disorders.

## Interoception Overview

Interoception refers to the process by which the nervous system senses, interprets, and integrates signals originating from within the body, providing a moment-by-moment mapping of the body’s internal landscape across conscious and unconscious levels [13]. Interoception has traditionally been considered to be a one-way street in which “bottom-up” signals traveling from the body to the brain cause sensation and elicit “top-down” regulatory responses when bodily homeostasis is disrupted [14••]. More recently, interoception has been adopted into the conceptual framework of Bayesian inference (a method of statistical inference in which new observations are used to continuously update or infer the statistical probability that a hypothesis/outcome may be true), based on the premise that afferent sensory input to the brain is constantly shaped and modified by the individual’s expectations [15–18]. Thus, interoception can be reconceptualized as a bidirectional process between the brain and the body, with feedback and feedforward loops that constantly update an internal model aimed at predicting and regulating future states of the body [19••]. Despite these theoretical advances and evidence supporting the idea that the brain and the body cannot be fully understood when studied separately, most explanatory neuroscientific approaches attempting to understand cognitive, emotional, and behavioral functioning in eating disorders have not integrated these two dimensions.

## Neurobiology of Interoception

The brain sits at the interface between the external world, which it samples through the exteroceptive senses, and the inner world of the body, which it accesses through interoceptive sensory channels. Interoceptive brain regions play primary roles in directly mapping the autonomic, chemosensory, endocrine, and immune systems, which relay information through peripheral nerves and direct neurochemical interfaces to the brainstem, hypothalamus, thalamus, and ultimately into cortical sectors including principally the insular and somatosensory cortices (for a detailed review see [14••]). The processing of information across these channels occurs in a hierarchical fashion, with multiple feedback loops starting in the autonomic nervous system and lower brainstem [20], providing a scaffold to delineate peripheral from central interoceptive dysfunction.

## Perceptual Inference and Predictive Processing

While distinct from interoception, perceptual inference is an overlapping construct referring to the process by which a person generates beliefs or explanations about the causes and effects of events occurring in the world [21]. Perceptual inferences are strongly influenced by expectations. They may be explicit or implicit and can rapidly change depending on the environmental context. Eating disorders are conditions that can be characterized by erroneous perceptual inferences—about appetitive, cognitive, sensory, affective, and interoceptive phenomena. Because they reflect beliefs, it is natural that these inferences can form the basis of subsequent disorder-specific behaviors (e.g., restrictive eating, binge eating, or purging).

Computational neuroscience has provided mechanistic insights into the underpinnings of causal inference in the nervous system. In predictive processing models [22], neurons transmitting predictions about sensory states communicate with neurons detecting deviations from those predictions (so-called “prediction errors”) to develop an explanation for the perceptual information received via a “generative model” [23]. Over time, when the observed information deviates from what is predicted, the generative model is updated through learning, and thus perception emerges from processing the external or internal world within the context of a prior model. In addition, the metacognitive evaluation of perceptual content plays a role in generating awareness states [24], and it is conceivable that abnormalities in the neural circuitry underlying metacognition (i.e., the awareness and understanding of one’s own thought processes) underpin aspects of eating disorder symptomatology [25], particularly in relation to interoceptive signaling.

## A Call to Reorient Interoceptive Investigations of Eating Disorders

Despite an early physiological focus on gut processing [26–33], progress in understanding the links between interoception and eating pathology has been limited by a predominant and selective reliance on self-report scales (see [34] and supplemental discussion in [13]). No experimental studies of interoception in eating disorders have utilized model-based analyses, which could formally test for altered predictive processing. Additionally, most existing studies focus narrowly on one feature (e.g., interoceptive accuracy) or one sensory channel (e.g., perception of heartbeats), ignoring the multifaceted interoceptive processes that may impact individuals with eating pathology. As mounting evidence suggests that individuals with eating disorders demonstrate maladaptive responses to food and eating-related sensations [35–37], new studies focused on gastrointestinal interoception are warranted. Such work would clarify associations of the neural circuitry central to the representation of gastrointestinal system with the affective and behavioral consequences of alterations within this system.

## Current Evidence of Gastrointestinal Dysfunction in Eating Disorders

Individuals with anorexia nervosa (AN) show an extreme ability to voluntarily ignore hunger/thirst signals to restrict caloric intake. This prolonged, severe food restriction, in turn, impacts the state of the gastrointestinal tract [38]. In clinical settings, patients with AN commonly report gastrointestinal complaints such as exaggerated fullness in response to small meals (i.e., postprandial fullness), early satiety, and abdominal pain [39•]. They also report bowel and bladder symptoms outside of mealtimes, such as fullness, bloating, and constipation [40–42], and frequently exhibit gastrointestinal disorders [43] as well as functional gastrointestinal disorders [44]. The physiology underlying these abnormal perceptions has not been examined extensively in laboratory settings. Most available studies have used naturalistic designs following inpatients during the refeeding process. For example, within inpatient settings, individuals with AN report premature fullness after eating small amounts of food [45–47]. Fructose-sorbitol bolus ingestion disproportionately provokes gastrointestinal symptoms [48], further suggesting a heightened visceral sensitivity in acutely ill inpatients. After short-term refeeding to promote weight gain and restore homeostatic balance to the gut, persons with AN continue to report exaggerated fullness [47], although to a somewhat lower extent [46]. These symptoms decrease substantially in the 6 months following inpatient treatment [49], raising the possibility that they reflect an indicator of successful treatment response.

Individuals with binge-eating disorder (BED) and bulimia nervosa (BN) engage in recurrent cycles of food overconsumption, and those with BN also engage in compensatory behaviors (e.g., restriction, self-induced vomiting, laxative misuse, excessive exercise). These behaviors influence and may be perpetuated by alterations in the gastrointestinal tract. Gastrointestinal complaints including bloating, nausea, and constipation are also common in BN and improve with treatment [50]. Childhood onset of gastrointestinal complaints is associated with earlier onset of bulimic symptoms[51]; however, it remains unclear whether gastrointestinal alterations precede or follow the development of binge eating and purging behaviors for most individuals. More laboratory studies have focused on the physiological underpinnings of these symptoms and characteristic behaviors in BN and BED than in AN. Results indicate disturbances in the perception of satiety [52] and reduced sensitivity to gastric distension [53] in BN and in BED [54•], which are associated with altered gastric motor function [54•] and abnormal gut hormone release (e.g., cholecystokinin [55]).

Finally, it has been noted that those with avoidant/restrictive food intake disorder (ARFID) may demonstrate elevated sensitivity in response to gastrointestinal symptoms; however, the extent to which heightened gastrointestinal sensitivity is driven by heightened sensory input from peripheral cues, or by heightened central sensory processes is currently unknown [11]. More work in this area is needed.

## Methodological Challenges and Solutions to Studying Gastrointestinal Interoception

Most attempts to examine the conscious perception of gastrointestinal sensations have used invasive approaches. These involve insertion of inflatable balloons into the esophagus [56–58], stomach [59], colon [60], or rectum [61–64], or direct gastric perfusion with chemical irritants [65]. While such mechanosensory approaches can be used to engage putative interoceptive cortical neural circuitry (i.e., insular and somatosensory cortices) [66–68], the invasiveness of these procedures is limiting, and they may provoke additional, confounding distress among eating disorder patients who have high levels of body image concerns.

Less invasive approaches exist but these, too, have certain constraints. For example, a water loading test involves ad-libitum ingestion of water until reaching a feeling of fullness [69]. This procedure can only be repeated once per testing session, hindering the ability to computationally model the perceptual processes underlying gastrointestinal sensation, and the protocol provides no information related to the processing of solid food. Other approaches have involved delivery of small amounts of tastants directly onto the tongue, such as sucrose solution [70], or milkshakes [71]. These approaches have contributed greatly to the understanding of the neurobiology of taste in eating disorders. However, they purposefully do not evoke the perceptual processes occurring in the gut after swallowing food, which represent most of the postprandial state.

Fortunately, recent developments provide a diversity of pharmacological and non-pharmacological approaches that are compatible with both experimental and observational methods for studying gastrointestinal interoception in eating disorders (Table 1). For example, we recently developed a minimally invasive probe for stimulating mechanosensory sensations in the stomach, via ingestion of a vibrating capsule [72]. Using a Bayesian computational modeling approach based on active inference, we could identify individual differences the evolution of prior beliefs during the task and their interactions with internal estimates of the reliability of gastrointestinal signals [73••]. Beyond the advantages of a minimally invasive method, notable benefits include the ability to administer repeated stimulations, thus facilitating sophisticated analyses. Several other commonly available approaches are relevant. Hydrogel capsules are a minimally invasive means capable of stimulating fullness sensations [74•]; the absence of caloric input associated with this approach allows for more naturalistic experiments separating the mechanosensory impact of gut distension from the caloric contents of a meal. Ingestible capsules for passive sensing of gut pressure, pH and temperature [75], and high-density electrogastrogram (EGG) arrays [76•] have been developed, which could facilitate laboratory as well as ambulatory (i.e., real-world) assessments of gut physiology in eating disorders across different stages of illness recovery. The discovery of a cortical “gastric network” in the brain using simultaneous analysis of resting EGG and brain fMRI recordings [77•] lays the groundwork for experimental studies testing the state-specific role of this network as a homeostatic regulator of food intake and of motivationally relevant hunger and satiety cues [78]. Glucagon-like peptide-1 (GLP1) receptor stimulation represents a noteworthy target for potential food craving modulation [79•] and is currently employed as an effective weight loss intervention in overweight or obese individuals [80]. A judicious and time-limited application of this approach as a research assessment tool in carefully screened eating disordered individuals may be warranted. For example, investigating the role of GLP1 agonism in modulating food craving and associated interoceptive neural circuitry in overweight individuals with BED would seem to be an appropriate approach, whereas similar studies in individuals with AN (or those engaging in prolonged fasting) might potentially increase harm by reinforcing food avoidance and propagating weight reduction. The motilin receptor agonist erythromycin has been shown to induce gastric contractions, hunger signals, and increased food intake [81], suggesting potential utility as an experimental probe of susceptibility to binge-eating behavior. The blockade of oral sucrose receptors [82] allows an opportunity to directly examine the influence of sucrose detecting cells in the stomach [83] in humans, particularly in relation to food cue processing in eating disorders. Finally, acute fasting represents a potent means of naturalistically modulating the strength of neurochemical hunger signaling with direct impacts on interoceptive neurocircuity [84••]. Collectively, these methods represent a diverse array of available tools for assessing the predictive value of individual differences in gastrointestinal interoception in eating disorders.

**Table 1:** Recent approaches relevant for investigating gastrointestinal interoception in eating disorders

Approach	Intended effect	Sensory transduction mechanism
Vibrating capsule (Vibrant Inc.) [72, 73••]	Provide mechanosensory stimulation to gut afferents, to measure conscious sensation	Unknown; likely stimulates mechanoreceptors (e.g., voltage-gated, PIEZOs)
Gelesis 100 hydrogel capsule (Gelesis Inc.) [74•]	Increase volume and elasticity of the stomach and small intestine contents, to generate the feeling of fullness	Unknown; non-aggregating cross-linked cellulose and citric acid particles increase in volume after absorbing water, creating a nearby mass effect
SmartPill™ Motility testing system (Medtronic Inc.) [75]	Measure pressure, pH and temperature throughout the GI tract, providing information on gastric emptying and GI transit time	None
High-density electrogastrogram [76•]	Estimate direction and speed of gastric slow waves following caloric stimulation	None
Gut-brain synchrony via resting state EGG-fMRI [77•]	Identify the “gastric network” defined by correlated activity between stomach and brain	Unknown
Semaglutide (Wegovy™) [79•]	Chronic weight management for overweight or obese individuals	Unknown; glucagon-like peptide-1 (GLP1) receptor agonist resulting in lower blood glucose levels. GLP1 receptors are located in the brain in interoception- and reward-relevant regions including the nucleus accumbens, ventral tegmental area, hypothalamus, and brainstem (nucleus tractus solitarius; NTS)
Erythromycin [81]	Induce gastric contractions, hunger signals, and food intake	Motilin receptor agonist
Gymnema sylvestre [82] (Sugarbreak Resist™ Strips/Spray)	Eliminate sweet taste perception	Block sucrose receptors in mouth or block intestinal sucrose absorption
16-h fast [84••]	Remove stomach contents and elicit hunger hormonal signaling cascade	Modulate food reward-motivation neurocircuitry

## From Expectation to Ingestion to Digestion: a Predictive Processing Account of Gastrointestinal Interoception

**Fig. 1:**
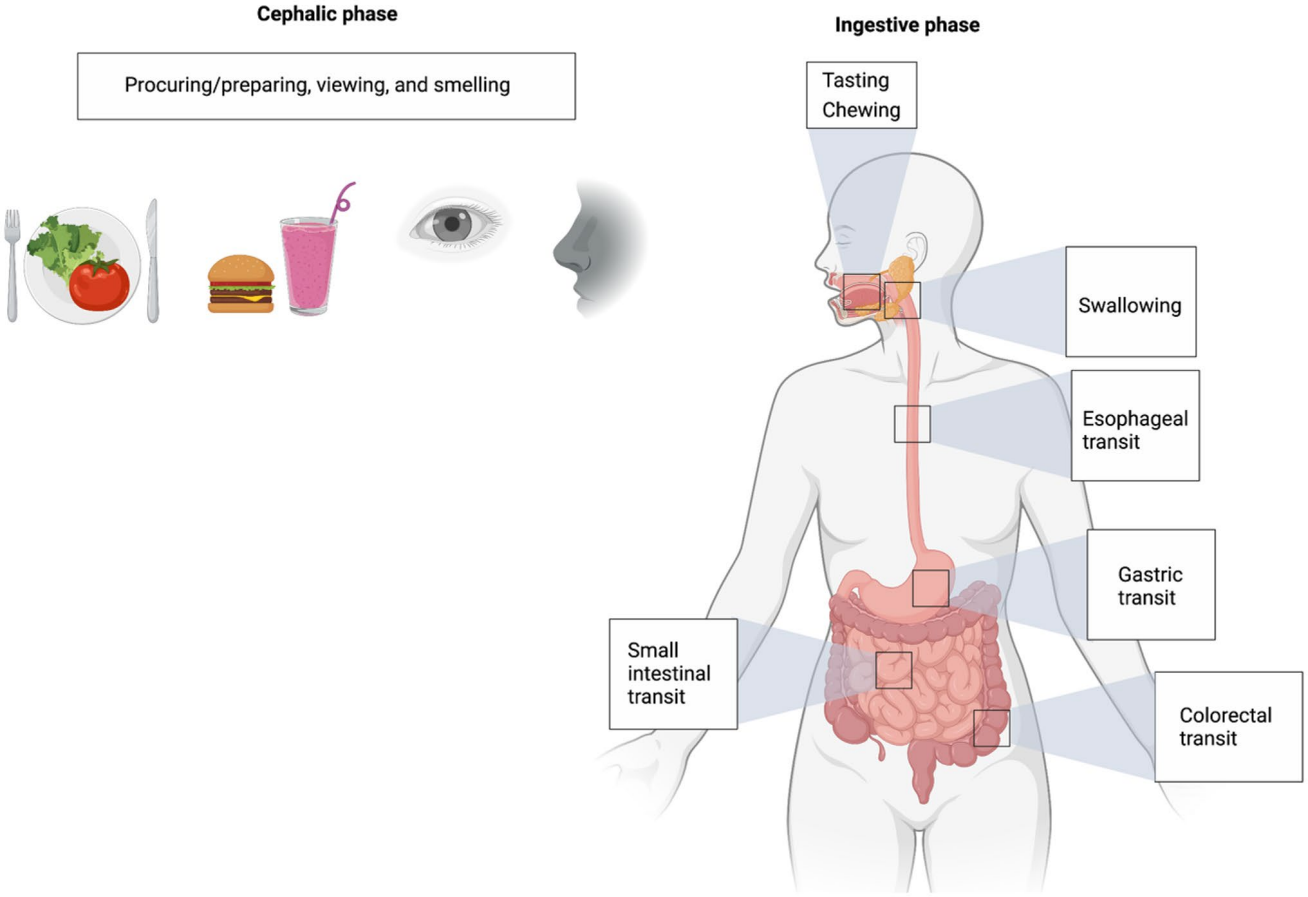
Phases of food consumption from foraging to expulsion. Starting with the cephalic phase, each phase involves predictive processing and likely has a dedicated gastrointestinal interoceptive neurocircuitry. Studies of interoception in eating disorders have preferentially focused on the cephalic rather than the ingestive phase of the digestive process. See text for details

Food consumption involves a series of anticipatory processing steps beginning with foraging during the cephalic phase followed by commencement of the ingestive process (Fig. 1). The cephalic phase includes processing via exteroceptive senses such as viewing and smelling. The motoric act of eating starts with tasting and chewing and is demarcated by swallowing, a key volitional checkpoint during which the ingested stimulus acquires the characteristics of an endogenous sensory signal [14••]. Subsequent steps with key relevance to interoception include esophageal transit, gastric filling and emptying, small intestine filling and emptying, and finally, colorectal filling and emptying. We suggest that abnormal interoception in eating disorders can manifest at each step via dysregulated bottom-up and top-down neural circuit interactions influenced by innate and developmental predisposing factors and various cognitive, valuative, and affective functions. In the following sections, we outline potential points throughout the alimentary tract for assessment and modulation of gastrointestinal interoception that may inform future research questions (Table 2) and, potentially, clinical applications targeting gastrointestinal interoceptive dysfunction in eating disorders.

**Table 2:** Future research questions

• What is the relationship between microbiome-host interactions and the trafficking of interoceptive signals in the gut? How does this crosstalk influence eating disorder expression?
• How do metabolic-, energetic-, and exercise-related genetic markers for eating disorders influence the neural circuits of interoception?
• How do gut predictions and prediction errors at each stage of food consumption influence the development and/or maintenance of eating disorder symptoms? Do increased gut prediction error signals promote restriction, while decreased gut prediction error signals promote binge eating and purging?
• How do the neural circuits of gastrointestinal interoception interact with those implicated in reward, emotion regulation, habitual behavior, and cognitive control?
• To what extent does the neurocircuitry underlying eating disorders overlap with that of functional gastrointestinal disorders?
• Which interoceptive targets within the gastrointestinal system show the most promise for early identification, prevention, or treatment of eating disorders?

### Procuring and Preparing

Exposure to food cues elicits cephalic phase responses (CPRs; i.e., physiological changes which prepare the body for digestion) [85]. Because foraging relies on the formulation of a hunger concept and an ensuing set of behaviors focused on obtaining food, from a predictive processing standpoint, we would argue that CPRs occur during the act of shopping, cooking, or simply ordering at a restaurant, and come with perceptible sensations. This stage thus requires the sensory predictive processing of hunger or desire and a future-oriented perspective that triggers a series of decision-making steps and ensuing actions. It is furthest from the receipt of the expected sensory signals that come from eating, and given the energy expenditures involved, requires the most in terms of motivation to maintain the necessary behaviors. The role of reward learning mechanisms has clear applications to this stage [86], and there is some evidence that individuals with eating disorders show goal-directed impairments in food and non-food related contexts [87, 88]. Yet, no studies have addressed the role of gastrointestinal interoception at this stage of food processing.

### Viewing and Smelling

Foraging is terminated by the arrival of food, when processing of the associated visual and olfactory sensory signals ensues. This cephalic stage of processing has been extensively studied in eating disorders, and abundant evidence indicates aberrant activation in interoceptive neural circuits in response to food images [36]. For example, individuals with AN show atypical relationships between stomach sensations and neural responses to food images in limbic and ventral striatal brain regions, both of which comprise key components of neurally-mediated interoceptive pathways that connect with the insular cortex [89]. Studies consistently report altered insula activation in response to food images in AN and BN, but the direction of these alterations has been inconsistent. As such, abnormal interoceptive processing in response to visual food cues may play a role in both disorders, but precisely how this processing is altered and how it might contribute to disordered eating remains unclear.

Though less studied than visual cues, responses to smells may be particularly important for gastrointestinal predictive processing. For example, parotid salivary secretion (the main component of appetitive salivary anticipation) depends upon exposure to olfactory signals [90], and olfactomotor responses to odors vary according to perceptions of pleasantness [91]. Hedonic ratings of food smells are associatively learned and depend upon the food- and hunger-related context [92]. Evidence that individuals with eating disorders show a heightened sensitivity to smells [93, 94] raises the possibility that interoceptive processing abnormalities triggered by olfactory stimulation play a reinforcing role.

### Tasting and Chewing

There is a longstanding debate regarding the extent to which taste is an interoceptive experience. Neurobiological arguments that taste is an interoceptive sense suggest that there are shared neural correlates of gustatory/taste experiences and interoceptive experiences, for example, within overlapping regions of the insula [95]. Taste signals are conveyed by similar channels as other interoceptive signals, and it has been suggested that defining gustatory neural representations could inform the understanding of interoceptive signals that are difficult to consciously access [96]. Conversely, studies of consummatory food reward suggest that gustatory signals may be more accurately conceptualized as exteroceptive [97, 98]. Debating the interoceptive versus exteroceptive categorization of taste perception is well outside the scope of the current review. However, recent studies have found that orosensory (e.g., taste) stimulation elicits overlapping and distinct neural activation patterns with interoceptive signals associated with gastric distension [99••]. As such, further investigation regarding the extent to which taste might represent a process that contributes to or maintains eating disorder psychopathology seems warranted.

In recent years, taste perception has garnered interest as a potential mechanism underlying food choice and eating behavior in individuals with eating disorders [100, 101]. Neuroimaging studies have shown anatomical alterations in women with eating disorders in brain regions centrally involved in taste and its valuation (e.g., medial orbitofrontal cortex, insula, striatum) [102]. Prior findings also suggest that women with acute restricting-type AN [103] and those recovered from AN or BN show aberrant anterior insula responses to sucralose [104, 105]. Women with restricting-type AN display a reduced ability to discriminate between sucrose, artificial saliva, or no solution, compared to healthy women and to women recovered from AN [106]. Additional findings suggest that individuals across the spectrum of eating disorders demonstrate abnormal ventral striatal-hypothalamic activation during a sucrose taste classical conditioning paradigm, with elevated prediction-error responses (violations of learned associations between conditioned visual and unconditioned taste stimuli) in AN [107] and reduced prediction-error responses in BN [108]. Altogether, although the taste-interoception debate has yet to be settled, taste-related deficits clearly play a role in eating disorder pathology.

Chewing is an understudied area of eating disorders, despite the fact that chewing and spitting behaviors are observed frequently in adolescent females [109]. Orosensory stimulation may be reinforcing in some individuals with eating disorders, as evidenced by the fact that social stress increases chewing rates in AN as compared to healthy individuals [110], and by modified sham feeding observations that women with BN sip more liquids [111] and women with AN sip less liquids [112] (independent of swallowing) than healthy individuals. More laboratory studies linking eating behaviors with gastrointestinal interoception are needed.

### Swallowing and Esophageal Transit

Deglutition, the action or process of swallowing, is a voluntary behavior that involves the triggering of a coordinated set of reflexes between the pharynx and upper and lower esophageal sphincter, which work together to transport food from the mouth through the esophagus. Under typical circumstances, the ingestion of food or liquid via swallowing marks a decision to move nutrients into the body as a metabolic means of maintaining homeostasis and survival, leading to a reflexive opening of the gastroesophageal sphincter for approximately 8 s [113]. Swallowing difficulties have been linked to posterior insular cortex stimulation [114], suggesting it may be a key node in the cortical neurocircuitry of this visceromotor action. We have previously argued that swallowing is a decisional checkpoint that serves as a pragmatic demarcation of the transition from object to interoceptive signal within the gastrointestinal system [14••].

Physical complications or disorders that interfere with this process can perturb the system and lead to distress, anxiety, and eating avoidance. Oropharyngeal dysphagia refers to difficulty with swallowing or transporting a food or liquid bolus from the mouth into the esophagus. Although commonly observed across eating disorders [115–117] dysphagia has been infrequently studied in relation to eating pathology. In ARFID, emerging evidence suggests that food avoidance and restriction develops in the context of medical conditions characterized by dysphagia or related fears of aversive outcomes associated with swallowing [118]. In AN, some individuals develop symptoms of dysphagia; this can be accompanied by the sensation of food getting stuck in the esophagus. For example, one case series suggested that dysphagia may occur in severe AN and can be treated using neuromuscular electrical stimulation in conjunction with swallowing therapy [119]. However, to date, no studies have examined the neurophysiological underpinnings of deglutition in eating disorders, suggesting further research in this area is needed.

Swallow studies provide a validated means of clinically assessing characteristics of swallowing, including oral sensation, chewing, salivation, and oromuscular coordination of food bolus transit. These tests use a radiopaque artificial food bolus for a contrast-enhanced fluoroscopic evaluation of swallowing [120]. They have revealed some evidence of abnormal swallowing sensations in AN [121] and could be used more broadly to interrogate esophageal interoception in eating disorders. Most studies of individuals with purging have focused on the reversal of the sensation of fullness in the stomach. However, assessing relationships among esophageal interoception, dysphagia, and self-induced vomiting behaviors (i.e., systematic reversals of esophageal transit) may pinpoint new mechanistic targets for treatment. In addition, many patients with BN report symptoms of esophageal acid reflux, but these may occur in the absence of abnormal esophageal or gastric mucosa [122]. Research investigating potential conditioned associations among swallowing sensations, negative affect, and urges to purge would help to delineate the regulatory influence of chronic vomiting on esophageal interoception.

### Gastric transit

The mechanosensory impact of gastric filling and emptying is a primary component of stomach sensation [123]. Altered postprandial gastric sensations are well-documented in eating disorders, and research to date suggests that these may arise from true (not just perceived) alterations in gastric function. In restrictive eating disorders, common reports of increased postprandial fullness and early satiety may relate to delayed gastric emptying and slowed orocecal transit [46, 124], and in BN and BED, delayed satiety may relate to reduced sensitivity to gastric distension [53, 54•]. To begin to elucidate how altered gastric predictive processing may maintain disordered eating, future studies could focus on integrating multilevel measures of gastric interoceptive accuracy (the correspondence between perceived gastric distension and objectively measured distension), beliefs about gastric interoceptive sensitivity, and subjective ratings of confidence in gastric interoceptive accuracy, with the correspondence or mismatch between confidence and accuracy [125]. Previous data suggest that large mismatches between objectively measured heartbeat perception accuracy and self rated sensitivity (conceptualized as “interoceptive trait prediction errors”) distinguish some individuals with autism spectrum conditions from healthy individuals, and that these “prediction errors” are inversely related to emotional sensitivity [126]. A similar mismatch between expectation and experience, but in the gastric domain, may maintain eating disorder symptoms. Thus, individuals with eating disorders may have abnormally and inaccurately strong expectations about situations that elicit gastric change (i.e., hyperprecise priors [127]), and they may have great difficulty adjusting these expectations in response to environmental changes. Characterizing abnormalities in gastric predictive processing could yield modifiable targets for novel eating disorder interventions.

### Small Intestinal Transit

Primary functions of the small intestine include the breakdown of semi-solid food in the proximal (duodenal) segment followed by nutrient and water absorption from the distal (jejunal and ileal) segments. Although not commonly investigated, there are both mechanoreceptors and chemoreceptors present in this region of the gut [128], and fasting as well as eating both influence the rate of filling/relaxation [129]. Mechanostimulation of the jejunum via balloon distension and chemostimulation via capsaicin (the active component of chili peppers) both induce feelings of pressure/fullness or cramping/pain and are typically localized in the same central abdominal region [130]. Thus, from an interoceptive standpoint, there are likely overlapping sets of sensory signaling processes at this segment of the gut. To date, there have been few experimental studies of small intestinal interoception in eating disorders, though one study suggested delayed small bowel transit times in individuals with AN [131], and a case study reported evidence of jejunal blockage in an individual with AN [132]. It is presently unclear to what extent abnormal processing of small bowel sensations plays a role in symptom generation in eating disorders.

### Colorectal Transit

The primary sensations associated with the distal end of the GI tract are linked to colorectal filling and emptying. In AN, high rates of defecatory disorders, constipation, and obstructive defecation syndrome suggest there may be slower than normal colonic transit timing [133]. These symptoms are supported by preliminary findings indicating delayed colonic transit times in individuals with AN who report chronic constipation [134]. Some individuals with AN also report abnormal sensation of rectal filling during anorectal manometry [134]. However, delayed colonic transit times normalize with refeeding [134], and data from a small group of patients with AN suggest that abnormalities in rectal sensation, internal anal sphincter relaxation threshold, rectal compliance, sphincter pressures, or expulsion patterns, normalize following weight restoration [135]. Additional work is needed to determine the extent to which sensorimotor rectal function is caused or maintained by interoceptive mechanisms (e.g., alterations in interoceptive network activation and increased or decreased perception of stimuli/sensations in the rectum) in eating disorders.

As previously noted, constipation is often reported by individuals with BN, and constipation and bowel hypofunction are common side-effects of laxative misuse [136]. These symptoms can, in turn, lead to increased sensations of bloating, promoting further eating-disorder behaviors. Medical complications often associated with chronic laxative or diuretic use (e.g., rectal prolapse) are also reported across eating disorders. The extent to which purgative eating disorder behaviors may be preceded by or contribute to aberrant colorectal interoception is unknown. Overall, there is presently great potential for the minimally invasive approaches (outlined in Table 1) to shed light on the causal neurobiological mechanisms related to gastric or colorectal transit of food signals and eating disorder symptoms.

## Non-Gastrointestinal Interoception in Eating Disorders

While the current review is primarily focused on gastrointestinal interoception, it is important to note that alterations broadly spanning other interoceptive domains (e.g., cardiac, respiratory, pain, soft cutaneous touch, temperature) have been postulated to contribute to a wide range of AN and BN symptoms: from extreme restriction despite starvation, out-of-control overeating episodes, and purging behavior to those that are less directly related to the gastrointestinal system such as body image distortion, anxiety, and alexithymia [137–140]. For example, a recent network analysis indicated that body mistrust, and not feeling safe in one’s body, most linked self-reported interoceptive awareness to severe eating disorder psychopathology [141]. These findings suggest that that mistrust of one’s interoceptive afferents in general is associated with eating disorder symptoms.

In the last 5 years, research focused on cardiac and respiratory systems in eating disorders has shed light on the abnormal anticipation and processing of interoceptive afferents that may underpin this mistrust. Women with current or past eating disorders show an altered brain response when attending to cardiorespiratory signals [142], and before and during perturbations in these systems. For example, women with a history of BN show insular hyperactivation during the anticipation of aversive breathing restriction and abnormally declining activation during this aversive interoceptive experience, whereas women with a history of AN show hypoactivation during the anticipation of breathing restriction and abnormally steep increases in activation during breathing restriction [143, 144]. These results support a potential role for interoceptive predictive processing, and specifically prediction errors, in both disorders. In addition, affective reactions to aversive respiratory sensations are elevated in AN and in BN [145•], suggesting that interoceptive signals may be not only “untrustworthy” but also more distressing. Notably, non-gastrointestinal interoception and gastrointestinal-related interoception likely interact to maintain eating disorder symptoms. For example, in AN, the anticipation of eating is associated with heightened sensations of heart palpitations, dyspnea, and anxiety, all of which decrease after meal completion [146, 147]; these studies raise the possibility of multisensory integration deficits in eating disorders via interactions between gastrointestinal and cardiorespiratory afferent signaling.

## Clinical Implications and New Horizons

Recent advances in the ability to modulate interoception provide new tools that may inform the development of individualized models and clinical interventions for eating disorders. For example, emerging evidence suggests that interoceptive exposure techniques targeting the gastrointestinal interoceptive cues (e.g., fullness, bloating) triggering anxiety and aversive states may attenuate symptom severity in adults and adolescents with eating disorders [148, 149, 150•]. Neuromodulation approaches such as vagus nerve stimulation (VNS) may have a physiological role in enhancing cardiovagal interoceptive processing [151], but auricular VNS does not modulate vagally-mediated heart rate variability [152], and the clinical applicability of non-invasive VNS for eating disorders is presently uncertain. Finally, reduced environmental stimulation therapy (aka floatation therapy) has received increasing study as a potential non-pharmacological anxiolytic. This approach is noteworthy in that it increases cardiorespiratory sensations while leaving gastrointestinal sensations unaffected [153]. After an early-phase trial showed this intervention to be safe, well tolerated, and associated with reduced anxiety and improved body-image symptoms in individuals with AN [154•], we are currently conducting a randomized efficacy trial. Despite initial hints of progress with respect to symptomatology, additional research is needed to clarify the extent to which these approaches effectively target interoceptive processes (especially predictive processing) that may contribute to or maintain pathological behaviors across the different phases of food consumption.

## Conclusions

In the current review, we have described a predictive processing approach to interrogating interoception at relevant points throughout the alimentary system. We have emphasized key conceptual definitions, methodological advances, and clinical implications that remain unexplored. There are still many gaps in our knowledge, but several existing tools could be easily applied to assess the role of gastrointestinal interoception in eating disorders. By revealing the basic neural circuitry involved in predicting and sensing gut feelings and defining those that are disrupted by eating disorders, these methods pave the way for a new generation of enterically focused assessments and clinical interventions for eating disorders.

## Footnotes

1 There are also emerging suggestions that the microbiome plays a role in eating disorders. However, the complexities of this “world within a world” combined with a limited knowledge of microbiome-host interactions at the level of interoceptive signaling renders this topic too early for us to review.
